# Advisory Committee on Immunization Practices Recommended Immunization Schedules for Persons Aged 0 Through 18 Years — United States, 2014

**Published:** 2014-02-07

**Authors:** Iyabode Akinsanya-Beysolow, Advisory Committee on Immunization Practices (ACIP), ACIP Child/Adolescent Immunization Work Group

**Affiliations:** 1 Immunization Services Division, National Center for Immunization and Respiratory Diseases, CDC

Each year, the Advisory Committee on Immunization Practices (ACIP) reviews the recommended immunization schedules for persons aged 0 through 18 years to ensure that the schedules reflect current recommendations for Food and Drug Administration–licensed vaccines. In October 2013, ACIP approved the recommended immunization schedules for persons aged 0 through 18 years for 2014, which include several changes from the 2013 immunization schedules.

*Recommendations for routine use of vaccines in children, adolescents, and adults are developed by the Advisory Committee on Immunization Practices (ACIP). ACIP is chartered as a federal advisory committee to provide expert external advice and guidance to the Director of the Centers for Disease Control and Prevention (CDC) on use of vaccines and related agents for the control of vaccine-preventable diseases in the civilian population of the United States. Recommendations for routine use of vaccines in children and adolescents are harmonized to the greatest extent possible with recommendations made by the American Academy of Pediatrics (AAP), the American Academy of Family Physicians (AAFP), and the American College of Obstetrics and Gynecology (ACOG). Recommendations for routine use of vaccines in adults are harmonized with recommendations of AAFP, ACOG, and the American College of Physicians (ACP). ACIP recommendations adopted by the CDC Director become agency guidelines on the date published in the* Morbidity and Mortality Weekly Report (MMWR)*. Additional information regarding ACIP is available at http://www.cdc.gov/vaccines/acip*.

For 2014, the figures, footnotes, and tables are not being published in MMWR; instead, a link to the CDC immunization schedule website is provided (http://www.cdc.gov/vaccines/schedules). This provides readers electronic access to the most current version of the schedules and footnotes on the CDC website. Health-care providers are advised to use both schedules and the combined footnotes together. Printable versions of the 2014 immunization schedules for persons aged 0 through 18 years also are available at the website in several formats, including portrait, landscape, and pocket-sized versions. Ordering instructions for laminated versions also are available at the website. “Parent-friendly” child and adolescent schedules are available at http://www.cdc.gov/vaccines/schedules/easy-to-read/index.html.

For further guidance on use of each vaccine included in the schedules, including contraindications and precautions to use of a vaccine, health-care providers are referred to the respective ACIP vaccine recommendations at http://www.cdc.gov/vaccines/hcp/acip-recs. In addition, changes in recommendations for specific vaccines might occur between annual updates to the childhood/adolescent immunization schedules.

These immunization schedules are approved by ACIP (http://www.cdc.gov/vaccines/acip/index.html), the American Academy of Pediatrics (http://www.aap.org), the American Academy of Family Physicians (http://www.aafp.org), and the American College of Obstetricians and Gynecologists (http://www.acog.org).

CDC’s National Center for Immunization and Respiratory Diseases (NCIRD) maintains the most current immunization schedules on the Vaccines and Immunizations pages of CDC’s website (http://www.cdc.gov/vaccines/schedules). If errors or omissions are discovered, CDC posts revised versions on those web pages. CDC encourages organizations that previously have relied on copying the schedules on their websites instead to use content syndication to consistently display current schedules. This is a more reliable and accurate method and ensures that the most current and accurate immunization schedules are on each organization’s website.

Use of content syndication requires a one-time step that assures that an organization’s website displays current schedules as soon as they are published or revised. Instructions for the syndication code are available at http://www.cdc.gov/vaccines/schedules/syndicate.html. CDC offers technical assistance for implementing this form of content syndication. Assistance from an NCIRD web team staff member is available by completing the e-mail form on the NCIRD web support page (http://www.cdc.gov/vaccines/about/contact/web_problem_form.htm).

Changes to the previous schedules† include the following:

- Several new references were added, including the 2014 adult immunization schedule (http://www.cdc.gov/vaccines/schedules) for vaccination recommendations for persons aged ≥19 years. Recommendations for persons who have been vaccinated before the minimum age/interval between doses of vaccine in a series also were added.
- Figure 1, “Recommended Immunization Schedule for Persons Aged 0 through 18 Years”:
  – Legend for the meningococcal conjugate vaccine row updated to reflect recommendation for use of MenACWY–CRM vaccine as early as age 2 months.
  – Pages 4 through 6 contain combined footnotes for each vaccine related to routine vaccination, catch-up vaccination,§ and vaccination of persons with high-risk medical conditions or under special circumstances.
- Standardized formatting used for footnotes for each vaccine to reflect the number of vaccine doses in a particular series.
  – Meningococcal conjugate vaccine footnotes updated to reflect recent recommendations for use of MCV4-CRM in high-risk persons aged 2 months and older.
  – Footnotes organized to reflect vaccine recommendations for each high-risk condition.
  – Influenza vaccine footnotes updated to provide guidance for dosing for children aged 6 months through 8 years for the 2013–14 and 2014–15 seasons.
  – Pneumococcal vaccine footnotes updated to provide guidance for vaccination of persons with high-risk conditions.
  – Hepatitis A vaccine footnotes updated to provide guidance for unvaccinated persons who are at increased risk for infection.
- Figure 2, Catch-Up Immunization Schedule:
  – *Haemophilus influenzae* type b (Hib) conjugate vaccine, pneumococcal conjugate vaccine, and tetanus, diphtheria, and acellular pertussis (Tdap) vaccine catch-up schedules updated to provide more clarity.

## Footnotes

§ For persons aged 4 months through 18 years who start late or who are more than 1 month behind in receiving recommended vaccinations.

